# Effect of Co-Doping on Thermoelectric Properties of *n*-Type Bi_2_Te_3_ Nanostructures Fabricated Using a Low-Temperature Sol-Gel Method

**DOI:** 10.3390/nano11102719

**Published:** 2021-10-15

**Authors:** Syed Irfan, Muhammad Aizaz Ud Din, Muhammad Qaisar Manzoor, Deliang Chen

**Affiliations:** 1 School of Materials Science and Engineering, Dongguan University of Technology, Dongguan 523808, China; 2 School of Materials and Energy, Southwest University, Chongqing 400715, China; aizazche@hotmail.com; 3 Department of Physics, Faculty of Engineering and Applied Sciences, RIPHAH International University, Islamabad 44000, Pakistan; greenpakistan2015@gmail.com

**Keywords:** Bi_2_Te_3_, sol-gel, electrical conductivity, Seebeck coefficient

## Abstract

In this work, a novel low-temperature double solvent sol-gel method was used to fabricate (Sm, Ce, Gd) and (Sn, Se, I) co-doped at Bi and Te-sites, respectively, for Bi_2_Te_3_ nanostructures. The phase-purity and high crystallinity of as-synthesized nanostructures were confirmed using X-ray diffraction and high-resolution transmission electron microscopy. The nanopowders were hot-pressed by spark plasma sintering into bulk pellets for thermoelectric properties. The spark plasma sintering temperature significantly affects the Seebeck coefficient and electrical conductivity of bulk Bi_2_T_e3_ pellets. The electrical conductivities of co-doped samples decrease with an increase in the temperature, but conversely, the Seebeck coefficient is linearly increasing. The power factor showed that the co-dopants enhanced the thermoelectric properties of Bi_2_Te_3_ nanopowders.

## 1. Introduction

Thermoelectric materials (TE) have gained significant interest in electricity demands, fossil fuel resource depletion, and climate problems due to the mutual transformation between thermal and electrical energy. According to the literature, 20% of the world’s total electricity generation is obtained from renewable energy resources, possibly increasing up to 39% by 2050. It will help to purify the atmosphere by removing CO_2_ up to 50% [1]. The dimensionless thermoelectric figure-of-merit (ZT) estimates the efficiency of thermoelectric devices, called ZT = (*S*^2^*σ*/*κ*)/T, where *S*, *σ*, *κ*, and T are the Seebeck coefficient, electrical conductivity, thermal conductivity, and the absolute temperature, respectively. The efficient thermoelectric device should have a high power factor (PF = *α*^2^*σ*) and low thermal conductivity [2,3].

The research interest in TE materials has been revived due to the quantum confinement effect and lower *κ* of low-dimensional materials than bulk materials with higher thermoelectric efficiencies at an absolute temperature [4]. Theoretically, the nanosized matrix can reduce the *κ* by creating several interfaces that cause strong scattering of a long-wavelength phonon. Simultaneously, nanofabrication can improve the thermoelectric properties in some cases [5,6,7]. The thermoelectric efficiency can be enhanced with the fabrication of nanostructured materials. Thus far, in improving the ZT value for the thermoelectric device, a bulk nano-structuring approach has made numerous improvements [8,9,10].

Recently, a significant increment in the ZT value has been reported for p-type Bi-Sb-Te alloy 1 to ≈1.4 by incorporating the nanostructure with the use of high energy ball milling mixed with hot pressing along with spark plasma sintering (SPS) [11,12]. At room temperature, the Bi_2_Te_3_-based devices have been studied as the most appropriate TE materials for energy conversion, particularly in bulk due to their sufficient constitute atomic composition, specific band structure, intrinsic low *κ*, and high carrier mobility [13]. The hexagonal, discrete, defect-free, and single-crystal Bi_2_Te_3_ nanoplatelets were fabricated by W. Lu et al. using a high-temperature organic solution technique [14]. Moreover, the nanotubes of Bi_2_Te_3_ were also synthesized via the solvothermal method by Y. Deng et al. Zhang et al. fabricated Bi_2_Te_3_ nanopores with small grain sizes using SPS and ball milling methods [15].

The nanopores demonstrated an orientation effect that showed its anisotropic electrical and heat conductivity [16]. The bulk Bi_2_Te_3_ materials were synthesized by melt spinning with SPS. The layered nanostructure of Bi_2_Te_3_ can effectively modify the carriers and phonon’s transport properties [17]. Moreover, the laminated nanolayers structure of Sb_2_Te_3_ and Bi_2_Te_3_, with a thickness of about 5 to 50 nm, were fabricated using the hydrothermal method by Y. Q Cao et al. [18]. In another work, the cathodic electrochemical deposition method was used to manufacture thin films of Bi_2_Te_3_ with optimized electrochemical parameters to obtain high-quality thin films. Due to this, a highly crystalline and morphological and compositionally uniform film was obtained [19]. Son et al. manufactured the ultra-thin Bi_2_Te_3_ nanoplates with a thickness of about −1 nm using the synthetic route [20]. Micro-assisted techniques were used to fabricate the nanosheets of Bi_2_Te_3_ by Z. Li et al. These nanosheets showed the average length of hexagonal sides of 320 nm, which was further verified using scanning and transmission electron microscopy [21]. In another experiement, the nanostructured materials were produced using the SPS process via the sintering of surfactant removed Bi_2_Te_3_ nanoplates. At 400 K, the maximum ZT was 0.62 for *n*-type nanostructured materials. The sol-gel method is a promising technique in the recently reported synthesis methods to fabricate Bi_2_Te_3_-based devices at room temperature. It is also environmentally friendly due to the loss of volatile components that can be curtailed at a lower temperature and are less expensive, multipurpose in fabrication and synthesized at large-scale production compared to vacuum processes [22,23,24].

This work used the novel double-solvent sol-gel method to synthesize the pure phase of Bi_2_Te_3_ nanoparticles at a low temperature. From the literature, no reports have been reported to synthesize Bi_2_Te_3_ nanoparticles using the described method. The thermoelectric properties of as-synthesized Bi_2_Te_3_ material were studied by varying the SPS temperature range from 350 to 450 °C. The results showed that the synthesis method affects the thermoelectric properties of pure Bi_2_Te_3_. Moreover, the co-doping of (Sm, Gd, Ce) and (Sn, Se, I) at Bi and Te-sites were used to observe the variation in the thermal behaviour of pure Bi_2_Te_3_.

## 2. Materials and Methods

### Fabrication of Co-Doped Nanostructures of Bi_2_Te_3_

The pure and co-doped Bi_2_Te_3_ nanostructures, abbreviated as Bi_2_Te_3_ (as BT), Ce_0.2_Bi_1.8_Te_2.97_Sn_0_._03_ (as CeBTSn), Sm_0.2_Bi_1.8_Te_2.9_I_0.1_ (as SmBTI), Sm_0.2_Ce_0.2_Bi_1.6_Te_3_ (as SmCeBT), Sm_0.2_Ce_0.2_Bi_1.6_Te_2.7_Se_0.30_ (as SmCeBTSe), Gd_0.1_Bi_1.9_Te_2.9_I_0.1_ (as GdBTI), and Sm_0.2_Gd_0.1_Bi_1.7_Te_2.9_I_0.1_ (as SmGdBTI) were synthesized using a low-temperature double solvent sol-gel method. The chemicals of Bi(NO_3_)_3_∙5H_2_O (98%, pure) and Te (99.8%, pure) powders were used as the starting precursors for pure Bi_2_Te_3_ and, where the co-dopants, (Gd(NO_3_)_3_∙6H_2_O (99.99%, pure), Sm(NO_3_)_3_∙6H_2_O (99.9%, pure), and Ce(NO_3_)_3_∙6H_2_O (99.5%, pure)) and (Se (99.99%, pure), Sn (99.5%, pure), and Iodine (99.99%, pure)) were used as an external element on to Bi and Te-sites, respectively. Ethylene glycol (C_2_H_6_O_2_) and acetic acid (C_2_H_4_O_2_) were added as double solvents. During the process, acetic acid acts as the catalyst to control the speed of hydrolysis and the solution’s concentration. In contrast, the C_2_H_6_O_2_ was used to preserve Bi and Te electronegativities during hydrolysis and its linearly structured molecule. Bi(NO_3_)_3_∙5H_2_O and co-dopants (Gd^3+^, Sm^3+^ and Ce^3+^) were added stoichiometrically, and dissolved in C_2_H_4_O_2_ and C_2_H_6_O_2_ with magnetic stirring for 90 min at 30 °C (Sol A).

Similarly, Te-powder and co-dopants (Sn^4+^, Se^4+^, and I) were mixed in C_2_H_6_O_2_ and C_2_H_4_O_2_ separately and magnetically stirred for 90 min (Sol B). The two solutions (Sol A and Sol B) were mixed together and magnetically stirred for 240 min at 35 °C. A blackish, partially dissolved solution (0.4 mol L^−1^) was obtained. After that, the combined solution was dried in a vacuum oven at 100 °C for a minimum of 12 h to form a gel. The dried homogenous amorphous powder was then sintered in a vacuum glass furnace in the Argon atmosphere at 240 °C for 6 h. After cooling down to room temperature, the BT powder was then ground to obtain the homogeneous nanoparticles. The bulk BT was prepared from SPS in a graphite die with a (diameter = 12.5 mm or 15 mm, thickness = 2 mm) temperature range of 350, 400, and 450 °C, under the pressure of 50 MPa in a vacuum.

#### Characterization Techniques

The crystallinity and phase purity of as-synthesized BT nanopowder was observed using X-ray diffraction (XRD, Cu-Kα radiation, Rigaku D/MAX-2550p diffractometer) (Bruker, Billerica, MA, USA). The 2*θ* range varies from the angle 10–80° with a step of 0.02° and scanning speed 4°/min. Scanning electron microscopy (SEM, Hitachi-S5500, Berlin, Germany), High-resolution transmission electron microscopy (HR-TEM, Titan Cubed Themis G201, FEI, Lexington, KY, USA), and energy-dispersive X-ray spectroscopy (EDS) investigated the surface morphology and elemental composition. The Archimedes rule estimated the density of bulk pellets prepared using SPS. The pellet’s mobility and carrier concentration were examined using the Hall effect measurement system (HL5500PC, Nanometrics). The *σ* and *S* value (rectangle bars shaped 2 × 2 × 15 mm pellet) were studied under a He atmosphere at various temperature points (SBA458, Nezsch, Germany). The thermal diffusivity (D) was studied using the laser flash diffusivity method (Laser Flash Apparatus LFA467, Netzsch, Germany). The thermal conductivity (*κ*) was estimated by using the formula, *κ* = *DC*_p_*ρ*, where *C*p is the specific heat capacity and *ρ* is the density. The *S*, *σ*, and *κ* were perpendicular to the pellet surface.

## 3. Results

### Phase Purity, Morphology, and Thermal Properties of Pure Bi_2_Te_3_

**Figure 1:**
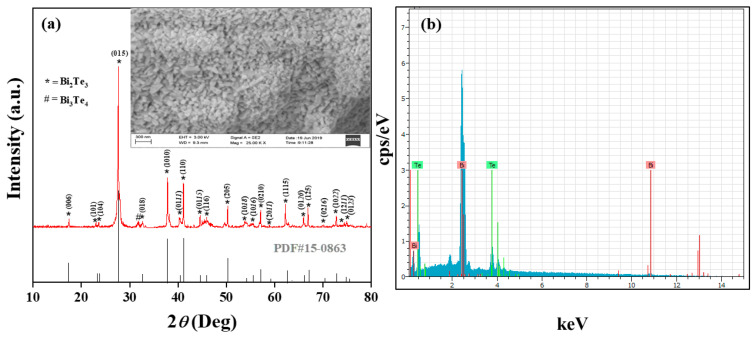
XRD patterns of as-synthesized BT using double solvent sol-gel method, (**a**) Pure BT, where inset shows an image of nanoparticles, (**b**) EDS analysis.

The X-ray diffraction pattern of as-synthesized BT powder was obtained with diffraction angle ranges from 2*θ*~10 to 80°, which confirmed that the major phases belong to BT, as shown in Figure 1a. The most intense diffraction peaks agree well with the rhombohedral structured BT (JCPDS 15-0863) [25]. A minimal number of impurity phases of Bi_3_Te_4_ were observed along with the pure phase of BT.

**Figure 2:**
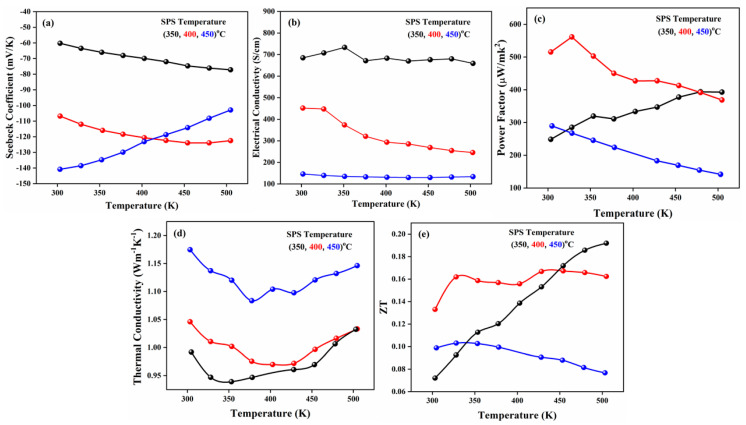
(**a**) S, (**b**) *σ*, (**c**) PF, (**d**) *κ*, and (**e**) ZT, of the *n*-type BT sample.

The partial solubility of Te-powder may be due to the fast evaporation rate of Bi compared with Te. EDS analysis confirmed the presence of Bi and Te in an as-synthesized sample, shown in Figure 1b. Moreover, the monocrystalline nature of pure BT is further verified by the HR-TEM images. To observe the thermoelectric properties of BT, SPS was used to prepare pellets at various temperature ranges. The three different SPS temperature ranges (350, 400, and 450) °C were used for sintering the pellets at 50 MPa pressure for 10 min. The sintering temperature range significantly affected the thermoelectric behavior of BT. From Figure 2a, BT has a negative *S* value, specifying *n*-type semiconductor behaviour. The *S* values for sample BT−350 °C showed an increasing trend (−60 to −76) mV/K with an increase in the temperature ranges from 300 to 500 K, and the same increasing trend for sample BT−400 °C (−107 to −123) mV/K.

However, a decreasing behavior in the *S* value (−141 to −104 mV/K) was observed for BT−450 °C, which confirmed that the further increase in operating temperature would decrease the *S* values. The *σ* values were slightly reduced from 690 to 670, 460 to 250, and 145 to 130 S/cm for the BT−350 °C, BT−400 °C and, BT−450 °C samples, respectively, shown in Figure 2b. The PF values in Figure 2c exhibit a mixed behavior of decreasing trend from 510 to 380 and 290 to 150 µW/mk^2^ for BT−400 °C and BT−450 °C samples, respectively, but an increasing trend, from 250 to 395 µW/mk^2^, was also observed for the BT−350 °C sample. In Figure 2d, an increased value of *κ* (1.17 Wm^−1^ K^−1^) was studied for sample SPS−450 °C at 300 K, but a decreasing trend was observed with the measurement temperature from 300 to 500 K. Figure 2e showed that the ZT continuously increased with the increasing temperature range from 300 to 500 K for the BT−350 °C sample, giving the optimized SPS temperature for BT-samples synthesized using the double solvent sol-gel method.

#### Phase Composition, Crystallinity, and Thermal Properties of Co-Doped Bi_2_Te_3_

**Figure 3:**
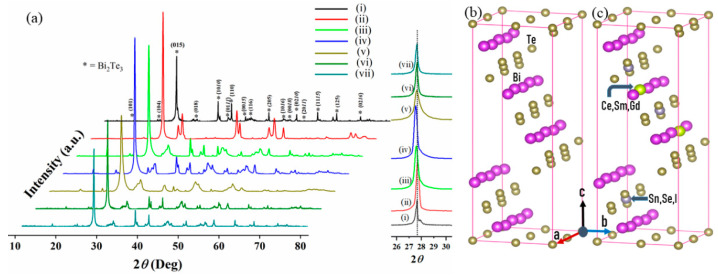
XRD patterns of (**a**) (i) Pure BT, (ii) CeBTSn, (iii) SmBTI, (iv) SmCeBT, (v) SmCeBTSe, (vi) GdBTI, and (vii) SmGdBTI, (where enlarged area showed the most intense peak at 2*θ*~27.55°), and the possible schematics for (**b**) Pure BT, and (**c**) co-doped BT.

The X-ray diffraction patterns of co-doped nanostructures of BT are shown in Figure 3. The most intense peak at an angle of 2*θ*~27.55 becomes sharper after incorporating the external atoms at Bi and Te-sites. From Figure 3, it can be seen that a slight shift of the most intense peak occurs, which confirmed the variation in the crystallite sizes after co-doping.

Moreover, a minor modification in the peaks of sintered nanopowder was due to the elimination of grain boundary defects after sintering. The broadening of the peak confirmed the successful replacement of the co-dopant atoms into BT lattice. The replacement of iodine atoms can be used as a connection between the two neighboring quintuple layers, therefore weakening the interface scattering. The doping of Sn showed the same lattice structure with a slight amount of unreacted Te-powder. The peaks broadening exhibited that the samples are nanosized. The XRD peak intensities of co-doped elements were inclined by the types of dopants and the degree of crystallization. These kinds of analyses outplace the capacity of this paper and would appeal quantum chemical calculations of bonding constraints, which will be the objective of our subsequent work. The possible schematics for the crystal structures of pure and co-doped BT nanostructures are shown in Figure 3b,c.

**Figure 4:**
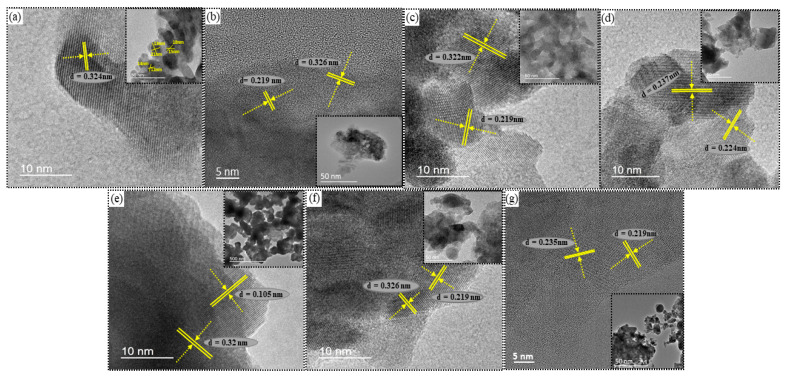
HR-TEM images for nano-powders of (**a**) Pure BT, (**b**) CeBTSn, (**c**) SmBTI, (**d**) SmCeBT, (**e**) SmCeBTSe, (**f**) GdBTI, and (**g**) SmGdBTI, (where inset area showed images at low-magnification).

The HR-TEM images offered more insight into the microstructural particulars, along with the size, morphology, and crystallinity of the as-synthesized BT nanopowder. The HR-TEM images of the pure and co-doped BT nanopowder are shown in the insets of Figure 4a–g, which showed that the particle size of pure BT varied from 13 to 50 nm. Figure 4a confirmed that the pristine BT has a single crystalline structure and exhibits that the spacing between adjacent lattice fringe is 0.324 nm.

**Figure 5:**
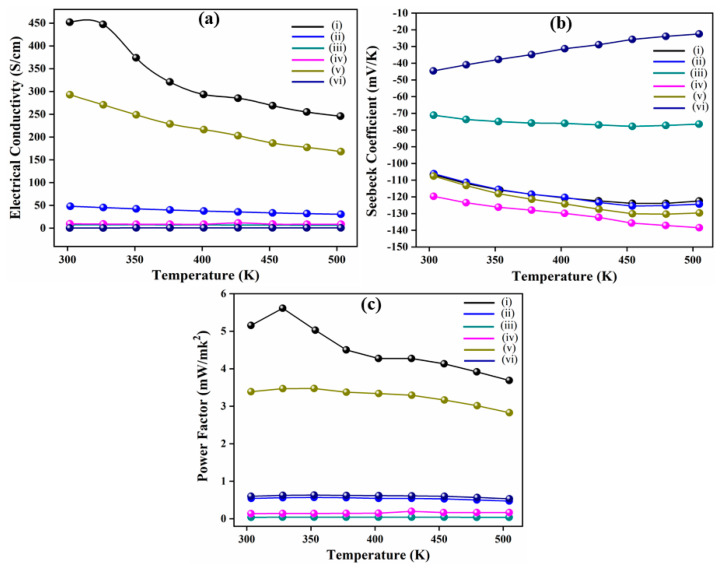
(**a**) *σ*, (**b**) *S*, and (**c**) PF, of the co-doped *n*-type BT sample, where (i) CeBTSn, (ii) SmBTI, (iii) SmCeBT, (iv) SmCeBTSe, (v) GdBTI, and (vi) SmGdBTI.

Figure 5a–c summarizes the electrical transport properties of the co-doped BT samples from room temperature to 530 K. The *σ* values of co-doped BT samples decrease monotonically with the increase in the temperature ranges. The CeBTSn sample significantly reduced the *σ* values from 450 to 250 S/cm; the same decreasing behavior was also observed for GdBTI and SmBTI from 295 to 172 and 50 to 40 S/cm, respectively, with the variation of temperature ranges. The pure and co-doped samples showed a decreasing trend for *σ* on an increased temperature range, which is the characteristic of degenerate semiconductors. However, a linearly slight increase in the *σ* values was also noticed for the SmCeBTSe and SmGdBTI, which confirmed that the Sm doping could help in improving the *σ* properties of pure and co-doped BT, synthesized using the double solvent sol-gel method, which may be ascribed to an increase in the charge carrier concentration in the BT with co-doping. In contrast, Figure 5b shows the temperature dependencies of *S* values. The co-doped BT samples exhibit *n*-type semiconductors due to their negative *S* in the measured temperature range from 300 to 530 K. The *S* values of the SmCeBTSe (absolute value) showed the highest *S* value amongst the other co-doped samples; it consistently increased from −120 to −138 mV/K.

Moreover, the *S* values showed a non-monotonic variation for the co-doped BT samples and changed smoothly from 300 to 473 K temperature range. The co-doping elements cause a shift of the *S* peaks, from 300 to 455 K, GdBTI, SmBTI, CeBTSn, and SmCeBT, respectively. This is a characteristic performance in thermoelectric materials; when increased, the incorporation of major external carriers did not allow the generation of the minor carriers and, therefore, an increase in the onset temperature of a bipolar effect [26]. On the other hand, the SmGdBTI sample exhibits a sharp decrease in the *S* value from −45 to −23 mV/K.

However, the *S* value of the co-doped samples is slightly higher than the pure BT samples synthesized at different SPS temperatures, as shown in Figure 5a–c. The reason may be that incorporating co-dopants into Bi generates several defects, which can mainly strengthen the scattering and direct it to a bigger scattering parameter *γ*, increasing the *S* value. Therefore, the co-doping elements can effectively increase the scattering, which increases the *S* value at elevated temperatures [27]. Moreover, the *S* values of the BT samples decreases with the increase in the temperature range higher than 480 K, which may be attributed to the rapid increase in the minor charge carriers. It is well known that for the degenerate semiconductors or metals, the *S* value is *α* *= (8π*^2^$k_{B}^{2}$*/3eհ*^2^)m*T(π/3n)^2/3^, where *k* is the Boltzmann constant, *հ* is the Plank contact, m* is the effective mass of the electron, and *T* is the absolute temperature (K). Therefore, the fluctuation in the *S* value may be caused by the possible variation of m* and *e.* From the above equation, the lower *S* value may be caused by the higher carrier concentration.

## 4. Conclusions

Pure Bi_2_Te_3_ and co-doped nanostructures, Ce_0.2_Bi_1.8_Te_2.97_Sn_0_._03_, Sm_0.2_Bi_1.8_Te_2.9_I_0.1_, Sm_0.2_Ce_0.2_Bi_1.6_Te_3_, Sm_0.2_Ce_0.2_Bi_1.6_Te_2.7_Se_0.30_, Gd_0.1_Bi_1.9_Te_2.9_I_0.1_, and Sm_0.2_Gd_0.1_Bi_1.7_Te_2.9_I_0.1_ were fabricated using the low-temperature double solvent sol-gel method. The X-ray diffraction technique was used to verify the phase purity of as-synthesized nanopowders, which was further confirmed using high-resolution transmission electron microscopy. The SPS temperature significantly affects the *S* and *σ* of bulk Bi_2_Te_3_ pellets. The *σ* values of co-doped samples decrease with an increase in the temperature, but conversely, the *S* is linearly increasing. The PF showed that the Ce and Sm dopants enhanced the thermoelectric properties of Bi_2_Te_3_ nanopowders. Therefore, the double solvent sol-gel method could be suitable for the low temperature synthesis of BT nanopowder, and the SPS-350 °C samples showed better thermal behavior for co-doped samples.

## Data Availability

All data in this study will be available from the corresponding author upon reasonable request.
